# Scientific Items

**Published:** 1883-04-20

**Authors:** 


### SCIENTIFIC ITEMS.

**Living a Double Life.**—Many men may be said to live two different lives, in one figurative sense or another; but it appears that such double existence may be a literal fact. A physician writing in a German medical journal relates the case of a woman who passes her life in two entirely distinct and alternating states. In one of them she can talk, but cannot swallow liquids; in the other she is able to swallow, but is deprived of all power of speech. While in one state she remembers perfectly all that has happened previously in that same state, but is totally oblivious of everything that occurred in the alternating existence. Lately a third state has developed, the external characteristic of which is a total paralysis of the right leg. All remembrance of this condition is also lost when once the patient has emerged from it.

A similar case has been recorded by Jessen. A young woman fell suddenly into a deep sleep, and upon awakening had lost all memory of past events. She was obliged to re-learn to read and write, and to make the acquaintance again of persons with whom she had formerly associated. After a few months she returned to her former condition. Thus she continued to alternate between the two states of existence, the change being always preceded by a deep slumber. Memory was retained only for events happening in the like state, and was lost for the alternating periods.—*Popular Science Monthly.*

**The Hygiene of Shoes.**—That the shoes we wear are seldom made of the proper shape has been often pointed out by scientific writers; but habit and fashion are not easily changed. The poor suffer more from this cause than the well-to-do, for cheap shoes are generally worse in pattern than more costly ones, and being clumsier and less flexible, cause greater distortion of the feet. Deformities of the feet and toes are especially frequent among the poor.

This matter was the subject of an able and interesting paper, read by Colonel Ziegler, Chief Surgeon of the Swiss army, at the Geneva Hygienic Congress. He stated that every year 800 recruits are rejected for malformation of the feet, resulting from badly fitting shoes. The foot is in reality a bow, so elastic that at every step it contracts and expands, lengthens and shortens, and a line drawn through the center of the great toe intersects the heel. Shoemakers do not give room enough for the lateral extension of the great toe, confining it until it is forced against the other toes, giving rise to inflammations, corns, ulcerations and sometimes true articular inflammations. Another evil is flat-footedness, whereby the arch of the foot is converted into a straight line, and prolonged walking rendered impossible.

Another cause of this defect is the carrying of heavy weights at an early age; but in most cases perfect shoes would restore the foot to its normal condition. A test of a perfect pair of shoes is that when placed together they should touch only at the toes and heels; the soles should follow the sinuosities of the feet, and to give room for their expansion should exceed them in length by fifteen or twenty millimeters.—*Popular Science Monthly.*

**Science and Christianity.**—It is too frequently the case that both the pulpit and the press arraign Science as being antagonistic to the Christian religion. Often are ministers, in their pulpit efforts, heard to say: "Science would have us believe there is no God; that the universe is the product of chance; that man was evolved from the monkey, and the monkey from the moneron; that there is no immortality—no heaven, no hell; *but Science is false!*" or words to that effect. Now, the fact is, Science teaches nothing of the kind; and all such assertions are but so many false accusations. Those who utter or print them, falsely arraign Science; and they do so because they fail to distinguish between Science proper and *false theories of Science!* Science, real and true, is one thing, and a theory of Science is another. A scientific *fact* is as much a truth of God as is the divine declaration that man must be born again before he can see the kingdom of God. But a theory of Science, based upon an imperfect knowledge of the scientific facts involved, is only a *theory*, and not by any means necessarily true, even though it be advocated by such eminent scholars as Spencer, Mill, Strauss. Kent, and Buckle; for when such men go beyond the absolute facts of Science, into the uncertain fields of speculation and imagination, they are as liable to err as are other fallible beings.

Science is knowledge systematized. Huxley says: "The *science* of any subject is the highest and most exact knowledge on that subject" The science of the material universe and its laws, so far as they have been ascertained by investigation, observation and experience. Hence, according to the generally received faith of believers, there can be no conflict between Science and Christianity, for both have the same author. The *facts* of the material universe are as much truths of God as are the moral obligations set forth in the Decalogue.—*Literary Microcosm.*

**One** by one the more precious metals are found deposited in this country, and in some cases, as in nickel, the unsuspected supplies prove greater in volume than the previous yield of all other countries combined. The latest of these discoveries is that of vanadium, which has been taken from an Arizona mine in larger paying quantities than ever before known.—*Ex.*

**The Electric Light.**—Paris physicians have demonstrated that the electric light is not at all injurious to the eye. Our parents used to tell us that looking at the lightning would make us blind. —*Exchange.*

A **Submarine Vessel.**—The submarine vessel now being constructed at Bucharest, it is claimed, will accomplish what no other submarine craft has ever been equal to. The plan contemplates a vessel capable of moving under water for twelve hours without any renewal of air.—*Ex.*

